# Identification of *Cytauxzoon felis* antigens via protein microarray and assessment of expression library immunization against cytauxzoonosis

**DOI:** 10.1186/s12014-018-9218-9

**Published:** 2018-12-29

**Authors:** Megan E. Schreeg, Henry S. Marr, Jaime L. Tarigo, Meredith K. Sherrill, Hilton K. Outi, Elizabeth H. Scholl, David M. Bird, Adam Vigil, Chris Hung, Rie Nakajima, Li Liang, Angela Trieu, Denise L. Doolan, Jennifer E. Thomas, Michael G. Levy, Mason V. Reichard, Philip L. Felgner, Leah A. Cohn, Adam J. Birkenheuer

**Affiliations:** 1 College of Veterinary Medicine, North Carolina State University, Research Building Room 464, 1060 William Moore Drive, Raleigh, NC 27607 USA; 2 College of Veterinary Medicine, University of Georgia, 501 D.W. Brooks Drive, Athens, GA 30602 USA; 3 College of Veterinary Medicine, University of Missouri, 1600 East Rollins, Columbia, MO 65211 USA; 4 College of Agriculture and Life Sciences, North Carolina State University, 2501 Founders Dr, Raleigh, NC 27607 USA; 5 School of Medicine, University of California Irvine, 1001 Health Sciences Rd, Irvine, CA 92617 USA; 6 QIMR Berghofer Medical Research Institute, 300 Herston Rd, Brisbane City, QLD 4006 Australia; 7 Australian Institute of Tropical Health and Medicine, James Cook University, 1 James Cook Dr, Douglas, QLD 4814 Australia; 8 Center for Veterinary Health Sciences, Oklahoma State University, 208 S McFarland St, Stillwater, OK 74078 USA

**Keywords:** Cytauxzoonosis, *Cytauxzoon felis*, Expression library immunization, Protein microarray, Piroplasmid, Vaccine

## Abstract

**Background:**

Cytauxzoonosis is a disease of felids in North America caused by the tick-transmitted apicomplexan parasite *Cytauxzoon felis*. Cytauxzoonosis is particularly virulent for domestic cats, but no vaccine currently exists. The parasite cannot be cultivated in vitro, presenting a significant limitation for vaccine development.

**Methods:**

Recent sequencing of the *C. felis* genome has identified over 4300 putative protein-encoding genes. From this pool we constructed a protein microarray containing 673 putative *C. felis* proteins. This microarray was probed with sera from *C. felis*-infected and naïve cats to identify differentially reactive antigens which were incorporated into two expression library vaccines, one polyvalent and one monovalent. We assessed the efficacy of these vaccines to prevent of infection and/or disease in a tick-challenge model.

**Results:**

Probing of the protein microarray resulted in identification of 30 differentially reactive *C. felis* antigens that were incorporated into the two expression library vaccines. However, expression library immunization failed to prevent infection or disease in cats challenged with *C. felis*.

**Conclusions:**

Protein microarray facilitated high-throughput identification of novel antigens, substantially increasing the pool of characterized *C. felis* antigens. These antigens should be considered for development of *C. felis* vaccines, diagnostics, and therapeutics.

**Electronic supplementary material:**

The online version of this article (10.1186/s12014-018-9218-9) contains supplementary material, which is available to authorized users.

## Background

*Cytauxzoon felis* is a tick-transmitted apicomplexan parasite that is the causative agent of cytauxzoonosis in domestic and wild felids in North and South America [1–8]. A closely related, genetically unique *Cytauxzoon* sp. has been identified in Europe [9–13], but has not been associated with classic cytauxzoonosis which is characterized by high mortality rates and massive proliferation and vascular dissemination of schizont-infected myeloid cells. Although no longer considered uniformly fatal in domestic cats, morbidity and mortality remain high for individuals presenting with acute cytauxzoonosis [14–19]. Even with treatment, which can cost thousands of dollars, mortality remains at least 40% [20]; a vaccine is not currently available. Following its initial discovery in Missouri in 1976, *C. felis* has since been detected in domestic cats in 19 states [6, 8, 21, 22] and in bobcats in two additional states [23, 24]. The prevalence of *C. felis* infections in enzootic regions of the USA has been reported at 6.2% [17], which is higher than the estimated prevalence rates of common infections like feline leukemia virus (2.26%) and feline immunodeficiency virus (4.2%, Companion Animal Parasite Council, https://capcvet.org/maps/#2017/all/felv/cat/united-states/, https://capcvet.org/maps/#2017/all/fiv/cat/united-states/). This pattern could be due to a combination of factors, including an increase in feline reservoirs, expanding range of competent tick vectors (*Amblyomma americanum* and *Dermacentor variabilis*), and increased clinician awareness and diagnosis.

Prevention of cytauxzoonosis appears to be the optimal control strategy, and currently depends either on application of prophylactic acaricides [25], or keeping cats indoors. However, in practice, these strategies are flawed. Despite recommendations to house cats indoors, an estimated 35–60 million pet cats are still allowed to roam outdoors in the United States [26, 27]. Furthermore, effective acaricide prophylaxis may be limited by lack of owner compliance or cost of acaricides, as a recent study indicated as few as 38% of cats presenting to a veterinary teaching hospital had not received any form of tick prevention [28]. We therefore propose that vaccination against *C. felis* would serve as a practical and cost-effective method of prevention. Because cytauxzoonosis is highly pathogenic, a vaccine that reduces morbidity and mortality, even if it failed to prevent infection, would be considered successful.

There is some evidence that domestic cats can develop a protective immune response against *C. felis.* Previous studies have demonstrated that cats that survive acute cytauxzoonosis are protected from clinical disease following subsequent challenge with a lethal dose of virulent *C. felis* [4, 29–31]. However, antigen discovery and vaccine development have been hindered by the inability to cultivate *C. felis* in vitro. To redress this we previously sequenced and annotated the *C. felis* genome, and undertook a search for vaccine candidates [32] via an approach known as “reverse vaccinology” [33]. The underlying premise is that each of the 4300 putative proteins that were predicted [32] could represent a potential vaccine candidate. To date, a single *C. felis* vaccine candidate has been identified and characterized using a single candidate gene approach [32]. Although identification of candidates in such fashion is conceptually straightforward, this approach is both labor-intensive and cost-prohibitive when considering the number of inferred protein-encoding genes present in the *C. felis* genome. As a more efficient strategy, we deployed a protein microarray to screen a large number of *C. felis* proteins for antigenicity. Protein microarray technology has been utilized as a strategy for identification and assessment of diagnostic and/or vaccine candidates for a variety of pathogens, including protozoal organisms [34–39] as well as fastidious organisms that have not been cultivated in vitro [40, 41]. Conceptually similar, an alternative, high throughput method for screening antigens as vaccine candidates in vivo is a DNA expression library immunization (ELI) approach. This entails immunization of individuals with a library of expression plasmids containing either a portion of or the entire genome of the infectious agent [42–44]. ELI has been used to identify protective antigens against a variety of protozoal infections in mice, including *Plasmodium*, *Leishmania*, and *Trypanosoma* species [42, 45–49].

Our goal was to develop a low-cost, fast-track approach for vaccine development that could be used for pathogens that are difficult to isolate/culture or for which there is limited funding available. The aims of this study were twofold. First, we described the utilization of a *C. felis* protein microarray for identification of antigens. Then we assessed the ability of these antigens to induce protection from clinical disease via ELI. Protein microarray successfully identified 30 *C. felis* antigens. However, ELI using these antigens failed to induce protection against disease or infection. Collectively, these data provide insight into the humoral immune response against *C. felis*, and help build a foundation for future vaccine development against *C. felis*.

## Methods

### *Cytauxzoon felis* DNA isolation, RNA isolation and cDNA synthesis

*Cytauxzoon felis* genomic DNA was extracted from leukoreduced blood (Purecell NEO Neonatal High Efficiency Leukocyte Reduction Filter for Red Cell Aliquots, PALL Corp., Port Washington, NY) using a commercially available kit (QIAamp DNA Blood Mini Kit Qiagen, Valencia, CA). RNA was extracted from liver tissue of a cat infected with *C. felis* using a commercially available kit (ZR Tissue and Insect RNA Kit, Zymo Research, Irvine, CA). Using this RNA as template, cDNA was synthesized with Taqman Reverse Transcription Reagents following the two step RT-PCR protocol according to manufacturer’s instructions (Roche, Mannheim, Germany).

### Identification and selection of *Cytauxzoon felis* genes/ORFs

Of the 4378 predicted *C. felis* proteins [32], we selected 864 as candidates (Additional file 1: Supplementary Data Set 1**)** to assess via protein microarray based on at least one of the following criteria: genomic synteny and/or sequence homology to antigenic proteins identified in related protozoal organisms; sequence homology to *Plasmodium* proteins that displayed heterologous reactivity against serum from cats infected with *C. felis* (data not shown); or the presence of putative features predicted in silico to increase antigenicity, including the presence of predicted signal peptides, transmembrane domains, and predicted antigenic sites (Signal P Server v.4.0 and TMHMM Server v.2.0 from the Center for Biological Sequence Analysis). Of the genes/ORFs encoding the 864 putative proteins, those that were 126–3804 bp in length were selected for amplification for inclusion on the protein microarray (768/864).

### PCR amplification of linear acceptor vectors (pXT7 and PVAX1)

We used high throughput homologous recombination to clone target genes for expression on the protein microarray (pXT7) and for incorporation into DNA vaccines (pVAX1). Linearized pXT7 was produced and amplified using previously described methods [50] with the following modification: Roche high-fidelity enzyme mix (2.5 U) and a 1X concentration of Expand high-fidelity buffer with MgCl_2_ was utilized (Roche, Mannheim, Germany).

Linearized pVAX1 (Invitrogen, Carlsbad, CA) was produced using identical methods as for pXT7. Primers were designed to amplify the linear acceptor vector and incorporate a Kozak sequence upstream of the cloning site and a stop codon downstream of the coding site (Additional file 2: Supplementary Table 1). The 25 μL reaction consisted of 1 ng of linearized pVAX1, 25 pmols of each primer (Forward: 5′-TAGATCCACTAGTCCAGTGTG-3′, Reverse: 5′-CCATGGTGGCCGAGCTCGGTACCAAGC-3′), 5 pmol of dNTPs, 1.155 U of Expand high-fidelity enzyme mix, and a 1X concentration of Expand high-fidelity buffer with MgCl_2_ (Roche, Mannheim, Germany). Thermal cycling conditions consisted of an initial denaturation step at 95 °C for 5 min, followed by 35 amplification cycles (94 °C for 20 s, 50 °C for 30 s, and 68 °C for 3.5 min) and a final extension step at 72 °C for 5 min (Techne Inc., Burlington, NJ).

### PCR amplification of *Cytauxzoon felis* genes/ORFs for protein microarray and DNA vaccine

For genes/ORFs included on protein microarray, we designed primers that included cassettes homologous cloning sites of the linearized pXT7 (Forward cassette: 5′-ACGACAAGCATATGCTCGAG-3′, Reverse cassette: 5′-TCCGGAACATCGTATGGGTA-3′); ORFs that were larger than 3000 bp were split into smaller fragments as needed for efficient amplification. Each 50 μL reaction contained 1 μL of genomic *C. felis* DNA template or cDNA, 50 pmol of each primer, 10 nmol of deoxynucleoside triphosphates (dNTPs), 2.5 U of Expand high-fidelity enzyme mix, and a 1X concentration of Expand high-fidelity buffer with MgCl_2_ (Roche, Mannheim, Germany). Thermal cycling conditions consisted of an initial denaturation step at 95 °C for 5 min, followed by 40–45 amplification cycles (95 °C for 30 s, 55 °C for 15 s, 50 °C for 15 s, and 68 °C for 3 min) and a final extension step at 68 °C for 10 min (Techne Inc., Burlington, NJ).

Similarly, for candidates included in the DNA vaccine, we designed primers with cassettes homologous to cloning sites of pVAX1 (Forward cassette: 5′-ACCGAGCTCGGCCACCATGG-3′, Reverse cassette: 5′-CACACTGGACTAGTGGATCTA-3′). Each 25 μL reaction contained 1 μL of genomic *C. felis* DNA template or cDNA (Additional file 2: Supplementary Table 1), 25 pmol of each primer, 5 nmol of dNTPs, 1.75 U of Expand high-fidelity enzyme mix, and a 1X concentration of Expand high-fidelity buffer with MgCl_2_ (Roche, Mannheim, Germany). Thermal cycling conditions consisted of an initial denaturation step at 94 °C for 5 min, followed by 40 amplification cycles (94 °C for 30 s, 55 °C for 15 s, 50 °C for 15 s, and 68 °C for 4 min) and a final extension step at 68 °C for 7 min (Techne Inc., Burlington, NJ).

PCR conditions were optimized for individual genes/ORFs as needed, and amplicons were confirmed by gel electrophoresis for correct size prior to proceeding to homologous recombination.

### High throughput homologous recombination cloning

All successfully amplified genes/ORFs were cloned into the appropriate plasmid expression vector (pXT7 for the microarray or pVAX1 for DNA vaccine) using a high throughput PCR recombination cloning method as previously described [50]. While the majority of plasmids cloned with pXT7 for the protein microarray were isolated without colony selection as described [50], colony selection was utilized for those plasmids cloned with pVAX1 for the DNA vaccine. Minipreps of clones were confirmed to have the correct insert via PCR using ORF sequence-specific primers and/or bi-directionally sequencing using T7 and BGH reverse primers; internal sequencing primers were designed and utilized as needed to obtain complete bi-directional sequencing (MCLAB, South San Francisco, CA).

### Protein microarray printing

The expression of successfully cloned genes/ORFs was carried out for 5 h via in vitro transcription-translation (IVTT) according to manufacturer’s directions (RTS *E. coli* HY 100 kit, biotechrabbit GmbH, Germany) with addition of 0.067 mM Brij-98. A selection of ORFs that were predicted to be soluble (n = 137) were expressed in duplicate and transferred to plates without addition of Brij-98. Crude proteins were printed immediately without purification on nitrocellulose-coated glass NOVA slides (Grace Bio Labs, Bend, Oregon 97702) using an OmniGrid 100 microarray printer (GeneMachines, San Carlos, CA). Controls spotted onto chips included IVTT reactions without plasmid and purified IgG, and protein expression was confirmed by probing for polyhistidine (clone His-1, Sigma) and hemagglutinin tags (clone 3F10, Roche) included within the PXT7 vector.

### Protein microarray probing

Serum was collected from 48 cats throughout the United States (20 cats PCR positive for *C. felis*, 20 cats PCR negative for *C. felis*, and 8 SPF cats that were PCR negative for *C. felis*). All 20 infected cats were known to have survived acute cytauxzoonosis. Four of these *C. felis* infected cats were known to be acutely infected and 4 were chronically infected.

Sera were diluted to 1:200 in Protein Array Blocking Buffer (GVS, Sanford, ME) with 10% (vol/vol) *E. coli* lysate and incubated at room temperature for 30 min. Blocking anti-*E. coli* antibodies in the serum samples with *E. coli* lysate helps reducing background reactivity against *E. coli* proteins from IVTT reactions. Arrays were rehydrated in Protein Array Blocking Buffer and incubated in sera overnight at 4 °C with constant agitation. Slides were then rinsed 10 mM Tris buffer (TBS, pH 8.0) containing 0.05% Tween-20 (TTBS), then incubated in biotin-conjugated, goat anti-cat immunoglobulin (anti-IgGfcc, Jackson Immuno Research, West Grove, PA) diluted 1/200 in blocking buffer. After rinsing the slides, bound antibodies were detected by incubation with streptavidin conjugated SureLightH P-3 (Columbia Biosciences, Frederick, MD). The slides were then washed three times in TTBS and three times in TBS followed by a final water wash. The slides were air dried after brief centrifugation and analyzed using a Perkin Elmer ScanArray Express HT microarray scanner (Waltham, MA).

### Protein microarray statistical analysis

The protein microarrays used here do not meet the criteria for required deposition under MIAME guidelines [51], and alternatives to standardize protein microarray results are in development to insure that all information can be easily interpreted (description of minimum information about a proteomics experiment [MIAPE] can be found here [52]). Intensities were quantified using QuantArray software utilizing automatic local background subtraction for each spot. ‘‘No DNA’’ controls consisting of *E. coli* IVTT reactions without addition of plasmid were averaged and used to subtract background *E. coli* reactivity from the unmanipulated raw data. All results presented are expressed as signal intensity. As previously reported [53], the ‘‘vsn’’ package in the Bioconductor suite (http://Bioconductor.org/) in the R statistical environment (http://www.R-project.org) was used to calculate seroreactivity. In addition to the variance correction, this method calculates maximum likelihood shifting and scaling calibration parameters for different arrays, using known non-differentially expressed spots. This calibration has been shown to minimize experimental effects [54]. We used raw values for the positive and negative controls to calibrate, and then normalize, the entire data set using the vsn package. Antigens were considered to be reactive if signal intensities were greater than the average signal intensity of the “No DNA” control spots, plus 1.5-times the standard deviation. Differential analysis of the normalized signals was then performed using a Bayes-regularized *t* test adapted from Cyber-T for protein arrays [55–58]. Benjamini–Hochberg p value adjustments were applied to account for multiple test conditions [59]. All p values shown are Benjamini–Hochberg corrected for false discovery.

### In silico analysis of antigens selected for inclusion in vaccines

A total of 33 different *C. felis* antigens printed on the protein microarray were chosen for inclusion two DNA vaccines (Table 1). Each candidate was assessed for amino acid sequence similarity to other *Piroplasmida* proteins via BLAST search (blastx) against *Theileria* and *Babesia* species genomes (PiroplasmaDB, http://piroplasmadb.org/piro/), *Plasmodium* species genomes (PlasmoDB, http://plasmodb.org/plasmo/), or all banked nucleotide sequences in the National Center for Biotechnology Information (NCBI, https://blast.ncbi.nlm.nih.gov/Blast.cgi).

**Table 1:** Characteristics of 33 antigens incorporated into the expression library vaccines

Candidate #^~^	Location in *C. felis* genome^!^	Selection criteria for microarray inclusion	Selection criteria for vaccine inclusion	Orthologue and/or synteny^%^	Signal peptide (Y/N)	Transmembrane domains	Antigenic sites
1	contig00088:95434-96586(−)	Antigenic features	Differential reactivity	Orthologous to conserved hypothetical protein (T. equi BEWA_039780)	Y	1	13
2	contig00034:39570-41574(+)	Antigenic features	Differential reactivity	None identified	Y	1	34
3	contig00195:8035-8711(+)*	Carboxy terminus of cf76: previously demonstrated antigenicity	Differential reactivity	Syntenic to conserved antigenic protein (T. parva p67, T. annulata SPAG-1, B. bovis BOV57)	Y	0	N/A
4	contig00109:22439-22690(+)	Antigenic features	Differential reactivity	None identified	Y	1	46
5	contig00259:2101-4206(−)	Antigenic features	Differential reactivity	Orthologous to conserved signal-peptide containing protein (T. equi BEWA_049350)	Y	0	24
6	contig00010:254-1016(+)	Antigenic features	Differential reactivity	None identified	Y	1	11
7	contig00147:58097-59225(−)	Antigenic features	Differential reactivity	None identified	Y	1	11
8	contig00411:10993-12107(+)^	Antigenic features	Differential reactivity	None identified	Y	1	13
9	contig00029:68709-70041(+)	Antigenic features	Differential reactivity	None identified	N	1	17
10	contig00237:32067-33665(+)	Heterologous *P. falciparum* reactivity, previously demonstrated antigenicity	Differential reactivity	Orthologous to conserved hypothetical protein (T. equi BEWA_027410, P. falciparum PF3D7_0522400)	N	0	N/A
11	contig00086:6446-7270(−)	Antigenic features	Differential reactivity	None identified	Y	0	12
12	contig00046:25575-26421(−)	Antigenic features	Differential reactivity	None identified	Y	1	10
13	contig00119:20915-22637(−)	Antigenic features	Differential reactivity	Orthologous to conserved hypothetical protein (T. orientalis TOT_020000805)	Y	9	14
14	contig00260:69016-71962(+)	Antigenic features	Differential reactivity	Orthologous to conserved hypothetical protein (T. equi BEWA_039370)	N	11	35
15	contig00052:839-1019(+)	Antigenic features	Differential reactivity	None identified	Y	1	2
16	contig00071:75760-77446(+)	Antigenic features	Differential reactivity	Orthologous to conserved hypothetical protein (T. orientalis TOT_040000434)	Y	9	20
17	contig00006:15286-16508(+)	Antigenic features	Differential reactivity	Orthologous to Theileria Sphingomyelin/lysocholinephospholipid- phospholipase C (T. equi BEWA_044200)	Y	0	19
18	contig00156:32608..32875(−)	Antigenic features	Differential reactivity	Orthologous to Babesia/Theileria/Plasmodium protein kinase domain containing protein, PK4 (T. equi BEWA_042970, P. falciparum PF3D7_0628200)	N	2	46
19	contig00195:6540-8711(+)*	Full length cf76: previously demonstrated antigenicity	Differential reactivity	Syntenic to conserved antigenic protein (T. parva p67, T. annulata SPAG-1, B. bovis BOV57)	Y	0	N/A
20	contig00214:29581-33385(+)	Antigenic features	Differential reactivity	Orthologous to Theileria/Babesia p-type ATPase (T. equi BEWA_037450)	N	8	52
21	ccontig00047:67240-68739(+)^+^	Antigenic features	Differential reactivity	Orthologous to conserved hypothetical protein (T. orientalis TOT_010000130)	Y	1	49
22	contig00093:7841..7964(+)	Orthology to *Theileria* antigenic protein (TaSE)	Differential reactivity	Orthologous to conserved antigenic protein (TaSE, Schizont Protein E, B. bovis secretory protein)	N	0	20
23	contig00130:22711..24075(−)	Antigenic features	Differential reactivity	Orthologous to conserved putative cysteine protease (T. equi BEWA_018680)	N	1	16
24	contig00137:10059-13245(−)	Antigenic features	Differential reactivity	Orthologous to conserved hypothetical protein (T. equi BEWA_041510)	Y	11	37
25	contig00088:68222-69173(−)	Antigenic features	Differential reactivity	Orthologous to conserved hypothetical protein (T. equi BEWA_011550)	N	5	9
26	contig00232:10901..11056(−)	Antigenic features	Differential reactivity	Orthologous to putative 50S ribosomal protein L17e (T. equi BEWA_024510)	Y	0	9
27	contig00433:34958-36380(+)	Antigenic features	Differential reactivity	Orthologous to conserved hypothetical protein (T. equi BEWA_053510)	Y	11	14
28	contig00147:21973-23388(+)	Antigenic features	Differential reactivity	Orthologous to conserved hypothetical protein (T. equi BEWA_041510)	Y	9	16
29	contig00145:9-1059(−)	Antigenic features	Differential reactivity	None identified	Y	0	17
30	contig00167:23417-24638(−)	Orthology to *Theileria* antigenic protein (TaSP, PIM)	Differential reactivity	Orthologous to conserved antigenic protein (TaSP, PIM)	N	3	7
31	contig00063:31158-31905(−)	Heterologous *P. falciparum* reactivity, orthology to Elongation Factor-1	Cross-reactive with near differential reactivity, previously studied in separate experiment	Orthologous to Elongation factor-1	N	0	N/A
32	contig00062:98497-98689(+)	Antigenic features	Increased reactivity in acute serum	None identified	N	9	14
33	contig00079:101222-102015(+)	Antigenic features	Increased reactivity in acute serum	Orthologous to conserved hypothetical protein (T. equi BEWA_022300)	N	3	12

~Candidates 1–30 were assigned numbers based on descending average seroreactivity to infected feline serum. Candidate 31–33 were chosen for vaccine inclusion for other reasons and randomly assigned numbers

!Location in *C. felis* genome refers to contig coordinates on PiroplasmaDB. Positive or negative sense are indicated in parenthesis

*Candidate 19 = Full length cf76, Candidate 3 = C-terminal region of cf76. In addition to being in CF-Library, these two candidate comprise CF-1

^Candidate 8 = Not included in CF-Library (unable to amplify)

+Candidate 21 = One portion of a larger ORF; indicated as “contig00047:66184-70054e2s1” in Additional file 1: Supplementary Data 1

%Gene ID of top orthologues (highest score/E-value) to newly identified *C. felis* antigens and/or *C. felis* antigens selected due to heterologous *P. falciparum* reactivity are listed in parentheses as pertinent. Gene ID listed refers to PiroplasmaDB or PlasmoDB nomenclature

*N*/*A* Not assessed

### Production of vaccines

Individual colonies confirmed to contain plasmids of interest were selected and grown in 150 mL LB/kanamycin (50 µg/mL) and were isolated according to manufacturer’s instructions (Zyppy Plasmid Maxiprep Kit, Zymo Research, Irvine, CA) or were commercially prepared (Genewiz, South Plainfield, NJ). Multiple maxipreparations were performed as needed to attain the desired final amount of plasmid. Plasmids were further concentrated by ethanol precipitation as necessary and were resuspended in endotoxin-free tissue culture grade distilled water (Thermo Fisher Scientific, Waltham, MA).

Two different vaccines were developed: “CF-Library” and “CF-1.” CF-Library consisted of 32 candidates, while CF-1 consisted of both the carboxy-terminal region and full-length *C. felis* cf76 (Additional file 2: Supplementary Table 1) [32]. Each vaccine dosage contained a total of 1 mg of DNA. CF-1 contained 500 μg each of C-terminal region cf76 and full-length cf76, while CF-Library contained 31.25 μg of each of the 32 plasmids.

### Animals

Eighteen, 11–18 month old, intact female purpose bred cats that were PCR negative for *C. felis* were obtained from a commercial supplier (Liberty Research, Inc., USA). Cats were cared for according to the principles outlined in the National Institutes of Health Guide for the Care Use of Laboratory Animals and were housed in AAALAC accredited facilities within sealed, climate-controlled isolation rooms with 12 h light/dark cycles. All animal use was approved by the University of Missouri Animal Use and Care Committee (protocol number 7909).

### Vaccine study design

Cats were randomly assigned to test and control groups, which are summarized in Additional file 3: Supplementary Fig. 1. Seven cats received vaccines: three cats (331, 623, and 638) received CF-1 and four cats (77, 308, 339, and 835) received CF-Library. Investigators were blinded to whether vaccinated cats received CF-Library or CF-1. Eleven cats did not receive vaccines: eight of these were infected with *C. felis* (positive controls for infection) and three were infested with *C. felis*-naïve ticks (negative controls for infection). Unvaccinated cats were also simultaneously enrolled in separate studies testing the efficacy of novel chemotherapeutics against cytauxzoonosis and the immune response to tick infestation and/or *C. felis* infection.

The schedule of vaccination and infection is summarized in Additional file 4: Supplementary Fig. 2. Cats within both vaccine test groups received three intramuscular (IM) injections. For the first two vaccinations (Day 6 and Day 30), cats received 1 mg of DNA (0.7 mL volume) via needle and syringe in the left (Day 6) or right (Day 30) cranial thigh (quadriceps femoris muscle). The third and final vaccination (1 mg of DNA in 0.25 mL) was administered on Day 50 in the caudal medial right thigh via VET JET transdermal vaccination system (Merial, Inc., Athens, Georgia). When using the VET JET device, cats were sedated with dexdomitor (15–20 ug/kg) and acepromazine (0.1 mg/kg) delivered intravascularly (IV).

### Challenge model

Cats were infected with *C. felis* 59 days after initial vaccination (Additional file 4: Supplementary Fig. 2) using tick transmission as previously described [25]. Uninfected cats were identically infested with *C. felis*-naïve ticks. For tick infestation, cats were anesthetized as described for transdermal vaccination above. Ticks were removed from cats 10–13 days post infestation, and the number of free and attached ticks as well as subjective engorgement of ticks were recorded for each cat (Additional file 5: Supplementary Table 2).

### Clinical evaluation

Once infected cats were inspected at least four times a day. Physical exams were performed daily, and attitude, heart rate, respiratory rate, and body temperature (measured rectally or by subcutaneous temperature chip (IPTT-3000, Bio Medic Data Systems, Seaford, DE)) were recorded 2–4 times daily.

Starting at 12 days post-infection and finishing at the resolution of clinical symptoms or death, blood was collected from each cat via the jugular vein or a subcutaneous venous access port (VAP; CompanionPort CP4, Access Technologies, Skokie, IL). Blood was collected into EDTA for complete blood count (CBC; Sysmex XT2000i V Automated Hematology Analyzer) and into red top tubes for serum biochemical profile (Olympus 400AUe Chemistry Analyzer). Frequency of hematologic and biochemical testing performed for each individual cat was dictated by severity of illness and discretion of attending veterinarian but was generally performed every-other-day, and peaks and/or nadirs of clinicopathologic parameters were compared.

### Therapeutic intervention

Supportive care and/or antiprotozoal therapy was initiated on a case by case basis at the discretion of attending veterinarian based on assigned treatment group (unvaccinated infected cats) or at the discretion of attending veterinarian if treatment was unassigned (vaccinated cats). Specific supportive therapies administered varied between cats and were tailored to the clinical needs of the individual (Additional file 6: Supplementary Table 3). Three different antiprotozoal treatment regimens were utilized in this study: two were experimental therapies that were administered to unvaccinated infected cats as a part of a separate study (Additional file 3: Supplementary Fig. 1). Experimental therapies included either Coartem [20 mg artemether + 120 mg lumefantrine/tablet (Novartis), 2 tablets BID for 3 days] or a combination of 4 oral antibiotics, referred to through the study as “4X Abx” (pradofloxacin 7.5 mg/kg q24 h, doxycycline 10 mg/kg q12 h, metronidazole 25 mg/kg q12 h, clindamycin 10 mg/kg q12 h until clinical improvement). For any cat that developed advanced cytauxzoonosis despite vaccination or experimental treatments, atovaquone (Mepron, GlaxoSmithKline) and azithromycin was administered as previous described [20].

### Assessment of infection, disease, and survival

Infection was confirmed by PCR amplification of parasite DNA (ITS-1 and/or 18S) as previously described [60–62] and if possible by identification of parasites via microscopic evaluation of blood smears. Cytauxzoonosis was defined as cats being febrile (body temperature ≥ 102.5 °F) and having at least one of the following three biochemical/hematological abnormalities: hyperbilirubinemia (> 0.3 mg/dL), neutropenia (< 2.5 × 10^3^ segmented neutrophils/µL), or thrombocytopenia (< 300 × 10^3^ platelets/µL). Survival rates of groups were compared using the Freeman Halton extension [63] of the Fisher exact probability test with significance set at p ≤ 0.05 (VassarStats, Poughkeepsie, NY).

### Assessment of humoral responses post vaccination and challenge

After the completion of the clinical trial portion of the vaccine pilot study, *C. felis* protein microarrays were probed as described above with sera obtained from cats from each test group. Sera were collected from vaccinated cats at 5–7 different time points throughout the study and were frozen at − 80 °C until protein microarray analysis (Additional file 4: Supplementary Fig. 2).

## Results

### Construction and quality assessment of *Cytauxzoon felis* protein microarray

Of the 864 *C. felis* genes/ORFs initially selected for analysis, 96 ORFs were excluded from further study due to small size and predicted minimal antigenicity and 48 ORFs were unable to be PCR amplified, leaving 720 genes/ORFs amplified for cloning. Of these, 15 were unable to be cloned, and 32 had an incorrectly-sized insert in the pXT7 plasmid, leaving a total of 673 proteins for printing on the protein microarray (93.5% cloning efficiency). Of these proteins, 633 proteins (94.07%) were efficiently expressed as determined by dual positive signals against the amino-terminal HIS and carboxy-terminal hemagglutinin (HA) tags.

### Identification of *C. felis* antigens and selection of vaccine candidates

**Fig. 1:**
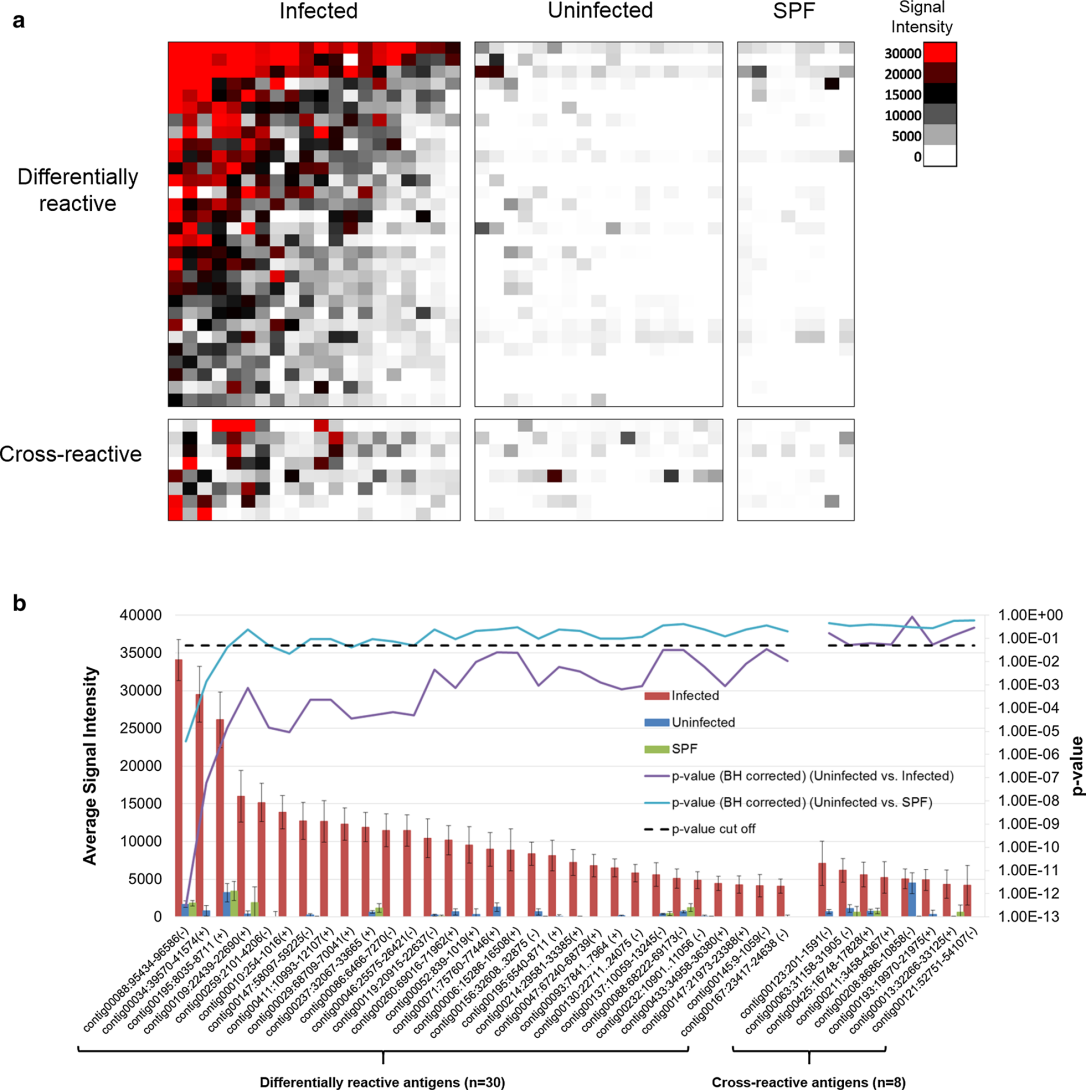
*Cytauxzoon felis* protein microarray identifies 30 differentially reactive antigens and 8 cross-reactive antigens. Serologic reactivity of antigens are depicted as a heatmap in **a**. Antigens are listed in rows while grouping of individuals are denoted in columns. Average signal intensity of individual antigens against serum from different groups are depicted in **b**

The antibody response profile to *C. felis* proteins in infected, uninfected, and SPF domestic cats is shown as a heatmap in Fig. 1. Of the total 673 genes/ORFs probed, a total of 38 (5.6%) were found to be seroreactive to feline serum. Of these, 30 were differentially reactive to serum from *C. felis* infected cats (p < 0.05) and 8 were cross-reactive to both infected and uninfected feline serum (p > 0.05). The number of infected and uninfected cats reacting to differentially reactive and cross-reactive proteins is summarized in Table 2.

**Table 2:** Summary of the number of cats with reactivity to differentially and cross reactive antigens

Order of reactivity	Location in *C. felis* genome	Number of infected cats with + reaction	Number of uninfected cats with + reaction
*Differentially reactive*			
1	contig00088:95434-96586(−)	20	2
2	contig00034:39570-41574(+)	19	1
3	contig00195:8035-8711(+)	20	3
4	contig00109:22439-22690(+)	16	1
5	contig00259:2101-4206(−)	17	0
6	contig00010:254-1016(+)	18	0
7	contig00147:58097-59225(−)	16	0
8	contig00411:10993-12107(+)	14	0
9	contig00029:68709-70041(+)	14	0
10	contig00237:32067-33665(+)	16	0
11	contig00086:6446-7270(−)	15	0
12	contig00046:25575-26421(−)	15	0
13	contig00119:20915-22637(−)	11	0
14	contig00260:69016-71962(+)	14	2
15	contig00052:839-1019(+)	10	1
16	contig00071:75760-77446(+)	11	3
17	contig00006:15286-16508(+)	10	1
18	contig00156:32608..32875(−)	14	1
19	contig00195:6540-8711(+)	11	0
20	contig00214:29581-33385(+)	10	0
21	contig00047:67240-68739(+)	11	0
22	contig00093:7841..7964(+)	11	0
23	contig00130:22711..24075(−)	12	0
24	contig00137:10059-13245(−)	6	0
25	contig00088:68222-69173(−)	7	0
26	contig00232:10901..11056(−)	8	0
27	contig00433:34958-36380(+)	10	0
28	contig00147:21973-23388(+)	9	0
29	contig00145:9-1059(−)	7	0
30	contig00167:23417-24638(−)	8	0
*Cross reactive*			
1	contig00123:201-1591(−)	5	1
2	contig00063:31158-31905(−)	10	1
3	contig00425:16748-17828(+)	6	1
4	contig00211:3458-4367(+)	6	0
5	contig00208:8686-10858(−)	7	7
6	contig00193:19970-21975(+)	8	1
7	contig00013:32266-33125(+)	5	0
8	contig00121:52751-54107(−)	3	0

Antigens are listed in order of descending average seroreactivity against *C. felis*-infected serum

Protein features of the 30 differentially reactive proteins include 21 proteins with a predicted signal peptide and 21 proteins with at least one and up to 11 predicted transmembrane domains (Table 1). All 30 proteins were predicted to have at least two and up to 52 antigenic sites (Table 1). Of these 30 proteins, four had been selected for placement on the microarray due to known orthology with antigenic proteins from related organisms (Table 1). One protein was selected due to homology to a conserved *Plasmodium* protein that previously showed reactivity against serum from *C. felis* infected cats (data not shown, Table 1). The remaining 25 proteins were selected for placement on the protein microarray primarily due to in silico predicted antigenicity, but on further investigation at least 15 of these proteins showed some degree of orthology to proteins from related organisms (Table 1). Ultimately, all 30 of the differentially reactive proteins were selected for incorporation into CF-Library (Table 1).

One cross-reactive protein was also selected for inclusion into CF-Library (Elongation Factor-1 orthologue). This protein was nearly classified as being differentially reactive (p = 0.052, Fig. 1), and was differentially reactive on a heterologous microarray screening sera from *C. felis* infected cats against *Plasmodium falciparum* antigens (Candidate 31, Table 1).

Of the infected feline serum samples, four were known to be collected while the cat was suffering from acute cytauxzoonosis, while four were known to be collected > 1 year after resolution of disease but while the cat remained persistently parasitemic. The antibody response profile to *C. felis* proteins in these samples were compared to assess for kinetic changes in serologic profile against *C. felis* (Additional file 7: Supplementary Fig. 3). A total of 52 proteins were found to be reactive (signal intensity > 1.5X No DNA standard deviation) to sera from either acute or chronically infected cats. No significant difference was seen (p > 0.05) for any proteins when comparing reactivity between serum of chronically or acutely infected cats. The majority of the proteins (n = 50) had a higher average reactivity against sera from chronically infected cats. However, two proteins had a higher average reactivity against sera from acutely infected cats (Additional file 7: Supplementary Fig. 3). These two proteins were also selected for inclusion in CF-Library (Candidates 32–33, Table 1).

### Vaccination and challenge

Of the 33 *C. felis* genes/ORFs selected for ELI, 32 were successfully cloned into the expression plasmid pVAX1 (97% cloning success rate; Table 1). Both developed vaccines (CF-Library and CF-1) were delivered successfully to cats (no significant volume loss during administration), with no adverse clinical effects observed post-vaccination (Additional file 4: Supplementary Fig. 2).

All cats infested with *C. felis* positive ticks met the criteria for both infection and cytauxzoonosis (Table 3), with the majority (n = 13/15 infected cats) exhibiting at least two of the three biochemical/hematological abnormalities that were considered disease-defining. No parasites were detected for cats infested with *C. felis*-naïve ticks and those cats did not develop cytauxzoonosis.

**Table 3:** Summary of notable vital signs, laboratory findings, and clinical outcomes for individual cats

Test group	Cat	Body temperature peak (°F)	Total bilirubin peak (mg/dL)*	Segmented PMN nadir (×10^3^/µL)*	Platelet nadir (×10^3^/µL)*	Infection confirmed	Cytauxzoonosis criteria Met	Supportive care	Antiprotozoal therapy	Survival
CF-library	77	104.3	1.9	3	57	Yes	Yes	No	None	Alive
	308	104.5	0.1	2.11	86	Yes	Yes	Yes	A&A	Alive
	339	104.4	0.6	5.62	41	Yes	Yes	Yes	A&A	Alive
	835	104.4	3.8	5.4	48	Yes	Yes	Yes	A&A	Alive
CF-1	331	104.4	1.5	2.83	112	Yes	Yes	Yes	A&A	Dead
	623	104.5	0.1	7.98	175	Yes	Yes	Yes	A&A	Dead
	638	104.2	2.7	1.3	41	Yes	Yes	Yes	A&A	Alive
Unvaccinated,	13	105.8	2.3	1.95	118	Yes	Yes	Yes	4X Abx + A&A	Alive
Infected	47	105.2	2	2.16	151	Yes	Yes	Yes	4X Abx + A&A	Alive
	59	105.3	3.4	1.66	51	Yes	Yes	Yes	Coartem	Alive
	84	103.4	0.2	3.89	281	Yes	Yes	Yes	Coartem	Alive
	577	104.7	4.8	4.15	98	Yes	Yes	Yes	4X Abx + A&A	Dead
	775	105.7	2.1	2.17	102	Yes	Yes	Yes	Coartem	Alive
	797	104.3	3.7	4.49	94	Yes	Yes	Yes	Coartem + A&A	Dead
	816	105.2	6.5	2.64	32	Yes	Yes	Yes	Coartem + A&A	Alive
Unvaccinated,	264	N/A	N/A	6.82	106	No	N/A	No	None	Alive
Uninfected	276	N/A	N/A	4.32	417	No	N/A	No	None	Alive
	880	N/A	N/A	6.96	271	No	N/A	No	None	Alive

Biochemical/hematological values outside of reference range are underlined for each individual

Segmented PMN = segmented neutrophil count, Band PMN = Band neutrophil count, A&A = atovaquone and azithromycin, 4X Abx = pradofloxacin, doxycycline, clindamycin, and metronidazole

*References ranges: Body temperature = 99.5–102.5 °F, Bilirubin: 0–0.3 mg/dL, Segmented neutrophils: 2.5–12.5 × 10^3^/µL, Platelets: 300–800 × 10^3^/µL

With the exception of one cat vaccinated with CF-Library, all infected cats received supportive care and antiprotozoal therapy (Table 3, Additional file 6: Supplementary Table 3). None of the uninfected cats became ill or required treatment (Table 3).

**Fig. 2:**
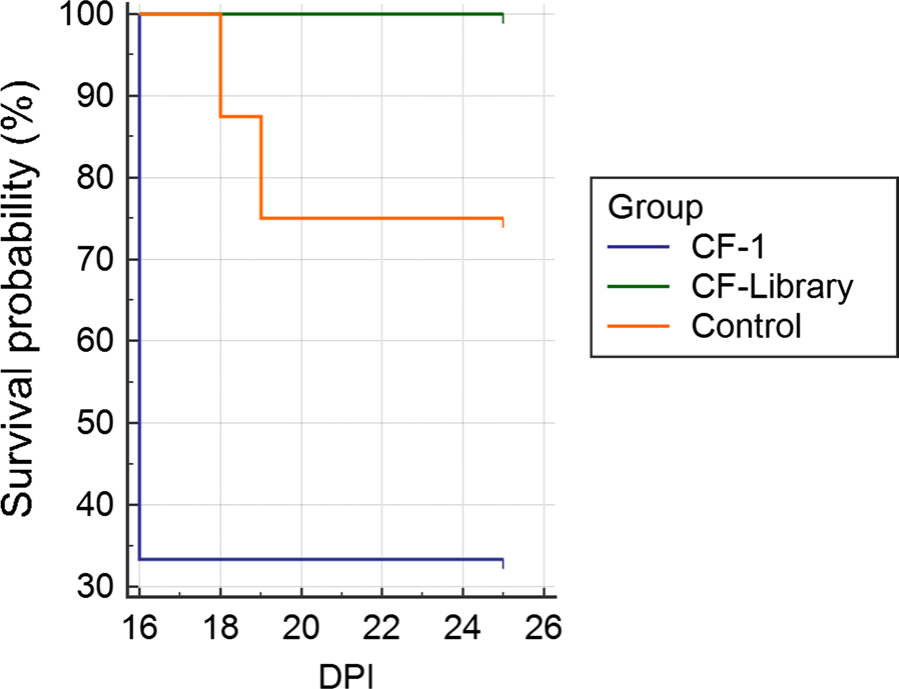
Cats vaccinated with CF-Library have a higher survival rate (100%) than other infected cats. Cats vaccinated with CF-1 had a 33% survival rate and unvaccinated, infected cats had a survival rate of 75%. Unvaccinated, uninfected cats also had a 100% survival rate (data not shown). *DPI* days post infection

Survival of individual cats is summarized in Fig. 2 and Table 3. Survival rate of the infected cats vaccinated against CF-Library was 100% (4 out of 4 cats survived). Survival rate of the infected cats vaccinated against CF-1 was 33% (1 out of 3 cats survived). Survival rate of the unvaccinated infected cats was 75% (6 out of 8 cats survived). Survival rate of uninfected cats was 100% (3 out of 3 cats survived). However, there was no significant difference between survival rates of groups (p > 0.05).

### Retrospective serological profiles of individual cats in response to vaccination and challenge

The serological responses of individual cats to vaccination varied (Additional file 8: Supplementary Fig. 4). Five cats (all cats vaccinated with CF-Library, one cat vaccinated with CF-1) developed a positive response to at least one antigen that they were vaccinated against prior to infection with *C. felis* (Table 4, Additional file 8: Supplementary Fig. 4). Two cats (two cats vaccinated with CF-1) failed to develop a positive response to any antigens vaccinated against prior to infection with *C. felis*, and in fact, these two cats only developed positive responses to antigens that they were *not* vaccinated against, including Candidates 4, 15, and 31 (Table 4, Additional file 8: Supplementary Fig. 4).

**Table 4:** Cats rarely seroconvert against antigens included in vaccine prior to infection with *Cytauxzoon felis*

Candidate	Gene/ORF	CF-1			CF-Library			
		331	623	638	77	308	339	835
19	contig00195:6540-8711(+)	N	N	N	Y—65	N	N	N
3	contig00195:8035-8711(+)	N	Y—30	N	Y—30	N	N	N
1	contig00088:95434-96586(−)	N	N	N	N	N	N	N
2	contig00034:39570-41574(+)	N	N	N	N	N	N	N
4	contig00109:22439-22690(+)	N	Y—30	N	N	N	N	N
5	contig00259:2101-4206(−)	N	N	N	N	N	N	N
6	contig00010:254-1016(+)	N	N	N	N	N	N	N
7	contig00147:58097-59225(−)	N	N	N	N	N	N	N
9	contig00029:68709-70041(+)	N	N	N	N	N	N	N
10	contig00237:32067-33665(+)	N	N	N	N	N	N	N
11	contig00086:6446-7270(−)	N	N	N	N	N	N	N
12	contig00046:25575-26421(−)	N	N	N	N	N	N	N
13	contig00119:20915-22637(−)	N	N	N	N	N	N	N
14	contig00260:69016-71962(+)	N	N	N	N	N	N	N
15	contig00052:839-1019(+)	N	N	Y—6	N	N	N	N
16	contig00071:75760-77446(+)	N	N	N	N	N	N	N
17	contig00006:15286-16508(+)	N	N	N	N	N	N	N
18	contig00156:32608..32875(−)	N	N	N	N	N	N	N
20	contig00214:29581-33385(+)	N	N	N	N	N	N	N
21	contig00047:67240-68739(+)	N	N	N	N	N	N	N
22	contig00093:7841..7964(+)	N	N	N	N	N	N	N
23	contig00130:22711..24075(−)	N	N	N	N	N	N	N
24	contig00137:10059-13245(−)	N	N	N	N	N	N	N
25	contig00088:68222-69173(−)	N	N	N	N	N	N	N
26	contig00232:10901..11056(−)	N	N	N	N	N	N	N
27	contig00433:34958-36380(+)	N	N	N	N	N	N	N
28	contig00147:21973-23388(+)	N	N	N	N	N	N	N
29	contig00145:9-1059(−)	N	N	N	N	N	N	N
30	contig00167:23417-24638(−)	N	N	N	N	N	N	N
31	contig00063:31158-31905(−)	Y—50	Y—30	Y—65	Y—65	Y—65	Y—50	Y—65
32	contig00062:98497-98689(+)	N	N	N	N	N	N	N
33	contig00079:101222-102015(+)	N	N	N	N	N	N	N

Candidates included in CF-1 are grouped at the top of the table (19 and 3). Individual cat numbers are listed below respective vaccine group. Y = Yes, N = No, number next to Y indicates day of seroconversion

Additionally, all seven vaccinated cats developed a positive response to Candidate 31 (Elongation factor-1) regardless of what they were vaccinated against, making it unclear whether the increase in seroreactivity against this candidate was *C. felis*-specific or not. When disregarding the positive responses against Candidate 31, only two cats developed positive responses against any other antigens they were vaccinated against. These included a positive response against either partial length cf76 (Cat 623, CF-1) or both partial and full length cf76 (Cat 77, CF-Library).

## Discussion

This study describes the construction and probing of a *Cytauxzoon felis* protein microarray for identification of antigenic proteins, the subsequent incorporation of those antigens into two expression library vaccines, and the evaluation of those vaccines to prevent clinical disease in cats challenged with *C. felis* infection. Using these methods, we were able to identify a pool of 30 *C. felis* antigens using the protein microarray, but were not able protect cats from developing cytauxzoonosis through ELI.

This study further validates protein microarray as a valuable tool for high throughput antigen discovery for organisms that are unable to be cultured in vitro. Prior to this study, only a single antigenic protein (cf76), which was identified by genome synteny with related organisms, had been characterized from a genome containing over 4300 putative proteins [32]. By use of the protein microarray, we were able to rapidly discover 30 new *C. felis* antigens (Fig. 1).

In this study only a portion (673/4378 = 15.4%) of the putative protein-encoding genes in the *C. felis* genome were analyzed. The percentage of differentially seroreactive proteins, 4.5% (30/673), was similar to percentages identified in other pathogens [34, 40, 64–67]. The pool of 673 proteins was chosen using methods to maximize the likelihood of identifying antigenic proteins. Studies evaluating the antigenicity of the remaining 3705 putative *C. felis* proteins should be the focus of future studies.

A limitation to this approach is that antigens identified by protein microarray are restricted to those that elicit a humoral immune response. Protection against *C. felis* is likely to require both a cell-mediated and humoral immune response, as the parasite resides intracellularly in the feline host. Unfortunately, high throughput methods for the identification of antigens that stimulate T-cells have only recently emerged, and are not cost-effective or widely available, particularly for use in a feline system [35, 68–70]. It has been suggested that proteins that induce a cellular immune response are also likely to be recognized by serum antibodies [36]. Therefore the antigens identified in this study should also be assessed for T-cell reactivity.

Unfortunately, ELI using antigens identified on the protein microarray in this study did not prevent *C. felis* infection or clinical disease (cytauxzoonosis). There are a number of possible explanations why ELI failed in this study. First, it is possible that failure occurred in any number of the steps required for a DNA vaccine to effectively confer immunity. This could include plasmid delivery into cells, expression of the antigens by the host cell, presentation of peptides on MHC molecules, and adequate stimulation of both innate and adaptive immune responses [42, 71]. Alternatively, vaccination could be ineffective due to inadequacies in dose, delivery route, vector, composition (lack of adjuvant), or vaccination schedule. Some of these deficiencies could be identified and overcome by assessing efficacy and immunogenicity of ELI (e.g. muscle biopsy to confirm plasmid expression, identifying uniform seroconversion) prior to challenge.

Second, it is possible that the antigens included in the vaccines were unable to induce a protective immune response against *C. felis*. First, recognition of a protein by antibodies does not definitively indicate a protective B-cell response [35, 36]. Furthermore, as stated previously, even if a B-cell response is generated, humoral immunity alone may not induce protection from disease. Additionally, antibodies against some of these proteins (e.g. Candidate 31, or elongation factor-1) may not represent a *C. felis*-specific immune response. Instead, it may represent production of cross-reactive antibodies against other pathogens (e.g. Coccidia), as it has been previously shown that serological cross-reactivity occurs between Coccidia and hemoprotozoan parasites [72, 73]. The increase in seroreactivity against these proteins (Additional file 8: Supplementary Fig. 4) could represent non-specific increases secondary to vaccination, which has previously been reported for other feline protozoan parasites [74].

We were attempting to develop a low-cost, fast-track approach for the development of a vaccine against a highly virulent feline pathogen that has never been isolated/cultured in vitro. Additional steps that should be taken in future studies include optimization and verification of the vaccination protocol prior to challenge, including verification of humoral immune responses by enzyme-linked immunosorbent assays or Western blotting.

One individual vaccinated with CF-Library (Cat 77) developed a comprehensive, albeit weak, serological response to vaccination, including a positive response to full length and the carboxy-terminal of cf76 (Additional file 8: Supplementary Fig. 4). Seven days post infection this cat’s antibody titers against all candidates decreased, suggesting that these antibodies may have been depleted during early infection (Additional file 8: Supplementary Fig. 4). Interestingly, this cat was the only individual in the study that only required minimal supportive care and no antiprotozoal therapy (Table 3). It is unclear if this was due to partial protection afforded by vaccination or due to this individual cat’s immune response.

## Conclusions

In conclusion, we identified 30 new *C. felis* antigens via protein microarray. These antigens could be considered for use in development of diagnostic tests or therapeutic interventions against *C. felis*. Bedside antigen detection tests are commonly used in veterinary medicine for rapid diagnosis of virulent disease [75], and monoclonal antibodies are emerging as a therapeutic tool against diseases afflicting veterinary patients [76]. Additionally, these antigens could still be considered for use in a vaccine against *C. felis* using a different vaccine platform.

## Additional files

**Additional file 1: Supplementary Data Set 1** Sequences of putative *Cytauxzoon felis* ORFs/genes selected for printing on protein microarray.

**Additional file 2: Supplementary Table** **1.** PCR and cloning information for vaccine candidates.

**Additional file 3: Supplementary Fig.** **1.** *Cytauxzoon felis* DNA vaccination pilot study design. A total of 18 cats were divided into four groups assessed in this study: three cats vaccinated with CF-1 prior to infection (red box), four cats vaccinated with CF-Library prior to infection (blue box), eight cats that were not vaccinated prior to infection (green box), and three cats that were neither vaccinated nor infected (gray box). The eight unvaccinated, infected cats were involved in a separate study testing the efficacy of novel chemotherapeutics against cytauxzoonosis. If it became evident that vaccines or chemotherapeutics were not halting the progression of disease, cats were additionally given atovaquone and azithromycin to attempt to prevent death; these cats are noted accordingly. A&A = atovaquone and azithromycin, 4X ABX = pradofloxacin, doxycycline, clindamycin, and metronidazole.

**Additional file 4: Supplementary Fig.** **2.** Timeline of vaccination, infection, and sample collection for vaccinated cats.

**Additional file 5: Supplementary Table** **2.** Additional clinical observations and information for individual cats.

**Additional file 6: Supplementary Table** **3.** Summary of supportive care administered to individual cats. Cats vaccinated with CF-Library received less overall supportive care.

**Additional file 7: Supplementary Fig.** **3.** Serological responses to *C. felis* antigens are increased during chronic infection. Serologic reactivity of antigens are depicted as a heatmap in (A). Antigens are listed in rows while grouping of individuals are denoted in columns. Average signal intensity of individual antigens against serum from acute and chronic groups are depicted in (B). The majority of antigens that had a higher average reactivity against serum from chronically infected cats (n = 50), but two antigens were more reactive against serum from acutely infected cats (n = 2, denoted with yellow boxes). These two antigens were incorporated into the CF-Library vaccine as Candidates 32-33.

**Additional file 8: Supplementary Fig.** **4.** Serological profiles of individual vaccinated cats to candidates throughout study. Arrays containing the 32 vaccine candidates included in CF-Library and in CF-1 were probed with sera samples from individual cats. Heat map shows normalized signal intensity with red strongest, white weakest, and gray intermediate. Rows denote 32 different candidates included in vaccines listed in descending order of reactivity; candidates 19 and 3 were included in CF-1, while all listed candidates were included in CF-Library. Results are organized by individual cats (identified by number at top), and survival status of each cat is indicated as “A” (alive) or “D” (dead). Individual columns within each cat’s array represent serum samples collected at different time points through study (labeled by the day in the study the sample was collected; refer to Supplemental Fig. 2 for timeline). There was no correlation between immunization protocol, individual reactivity, and survival for most cats, with the exception of Cat 77, who had widespread yet weak reactivity against all candidates in the CF-Library vaccine prior to infection and subsequently had milder disease and did not require supportive care or antiprotozoal therapy.
