# Supplementary material for: Identification of Cytauxzoon felis antigens via protein microarray and assessment of expression library immunization against cytauxzoonosis
Source: Clin Proteomics. 2018 Dec 29;15:44. doi: 10.1186/s12014-018-9218-9 (PMC6310948; doi:10.1186/s12014-018-9218-9)
Supplement: Supplementary file 2 — Additional file 2: Supplementary Table 1. PCR and cloning information for vaccine candidates. [file 12014_2018_9218_MOESM2_ESM.pdf]

Supplementary Table 1. PCR and cloning information for vaccine candidates.

Candidate	Location in *C. felis* Genome	Forward Primer	Reverse Primer	Size (BP)	PCR Template
1	contig00088:95434-96586(-)	ACCGAGCTCGGCCACCATGGTGATGAGACTTGTAGGTAAAGT	CACACTGGACTAGTGGATCTATGATCCCGTACCTTGTGAAA	1152	DNA
2	contig00034:39570-41574(+)	ACCGAGCTCGGCCACCATGGGTCGTAGACGTATTGT	CACACTGGACTAGTGGATCTATTGTTGTGTGGTACGTAGAG	2004	DNA
3	contig00195:8035-8711 (+)*	ACCGAGCTCGGCCACCATGGAAGAACATTCAATAGCT	CACACTGGACTAGTGGATCTAAACTAGTGTTAATGATAAC	832	DNA
4	contig00109:22439-22690(+)	ACCGAGCTCGGCCACCATGGTTGTTGGTATTTTAATCGG	CACACTGGACTAGTGGATCTATTGTCTTGGGAGTGGGATTT	249	DNA
5	contig00259:2101-4206(-)	ACCGAGCTCGGCCACCATGGATCTAGGGTACATAAACGC	CACACTGGACTAGTGGATCTAAATCGATTGAATTAATGTTA	2106	DNA
6	contig00010:254-1016(+)	ACCGAGCTCGGCCACCATGGTGATGAAATCGTTCACATTTCT	CACACTGGACTAGTGGATCTAGATCTTAAGTGTGAAAAGGA	762	DNA
7	contig00147:58097-59225(-)	ACCGAGCTCGGCCACCATGGTGATGAATTTTAAGGTAGTTGG	CACACTGGACTAGTGGATCTATGTAGTTTGTCTCTGTTGTT	1128	DNA
8	contig00411:10993-12107(+)^	ACCGAGCTCGGCCACCATGGTGATGCAAGAGAATAAGTTATT	CACACTGGACTAGTGGATCTATTGAGCAGGGGTAGAAGCGC	1114	DNA
9	contig00029:68709-70041(+)	ACCGAGCTCGGCCACCATGGTGATGCTAGTGTCTAATGACTA	CACACTGGACTAGTGGATCTATACACCCCCAGGATATAAAC	1332	DNA
10	contig00237:32067-33665 (+)	ACCGAGCTCGGCCACCATGGTGATGTTACAGTATATTGTACATCAATT	CACACTGGACTAGTGGATCTAAATGAATTCCAAGTCATCATTAGATGG	1732	DNA
11	contig00086:6446-7270(-)	ACCGAGCTCGGCCACCATGGATTTTTCAATATTTGT	CACACTGGACTAGTGGATCTAACTTAAAATAGATACTAGGA	804	DNA
12	contig00046:25575-26421(-)	ACCGAGCTCGGCCACCATGGTGATGACCATGAGTTACAGTAA	CACACTGGACTAGTGGATCTATGATGTACCTGTAGGTTTCG	846	DNA
13	contig00119:20915-22637(-)	ACCGAGCTCGGCCACCATGGTGATGAAGAATGAGACATATAA	CACACTGGACTAGTGGATCTAATTATATTCTGCTTTGCCCT	1772	DNA
14	contig00260:69016-71962(+)	ACCGAGCTCGGCCACCATGGAATCCTATTTTAGGAA	CACACTGGACTAGTGGATCTAACCATCTTCCACAAATAGTC	2946	DNA
15	contig00052:839-1019(+)	ACCGAGCTCGGCCACCATGGCATTTGATCTATTACA	CACACTGGACTAGTGGATCTATTCATTTTTGTTGTGATCGT	180	DNA
16	contig00071:75760-77446(+)	ACCGAGCTCGGCCACCATGGTGAAGCAACAATTATTAAGAGT	CACACTGGACTAGTGGATCTAAACATTGCAAGCTGAATATT	1686	DNA
17	contig00006:15286-16508(+)	ACCGAGCTCGGCCACCATGGTGATGAGACTATTACTCTTCAT	CACACTGGACTAGTGGATCTATTGTATTTTACAGAAATCAT	1224	DNA
18	contig00156:32608..32875 (-)	ACCGAGCTCGGCCACCATGGTGAGGCCTACCAGAAGTGATAA	CACACTGGACTAGTGGATCTAATCACTTAGTAAATGAGCTT	270	DNA
19	contig00195:6540-8711 (+)*	ACCGAGCTCGGCCACCATGGAATTTTTATTAATGTTTGTGGTGC	CACACTGGACTAGTGGATCTAAACTAGTGTTAATGATAAC	2277	DNA
20	contig00214:29581-33385(+)	ACCGAGCTCGGCCACCATGGTGATGACCAATGAAGATCATGG	CACACTGGACTAGTGGATCTAGTTTAGGTGTTTTTTCATTC	3804	DNA
21	ccontig00047:67240-68739(+)	ACCGAGCTCGGCCACCATGGTGATTGTTACCAAGGCACCAGA	CACACTGGACTAGTGGATCTATGGTATAGATGGTTTAGTAA	1499	DNA
22	contig00093:7841..7964 (+)	ACCGAGCTCGGCCACCATGGTGAGGAAATTGAGGGACTCTGG	CACACTGGACTAGTGGATCTAATTAGATTCATTTTCTTTGT	126	DNA
23	contig00130:22711..24075 (-)	ACCGAGCTCGGCCACCATGGTGATGTTGATTGAGGAGCCACT	CACACTGGACTAGTGGATCTAATCTGAGAATGAGTTGAATG	1365	DNA
24	contig00137:10059-13245(-)	ACCGAGCTCGGCCACCATGGATGGTTTGTTGGGTAT	CACACTGGACTAGTGGATCTATATGTTTTGTGGTGGATCTA	3186	DNA
25	contig00088:68222-69173(-)	ACCGAGCTCGGCCACCATGGTGATATTGGATGTTGAGCCTAA	CACACTGGACTAGTGGATCTATACTATTTCATATGCACCTT	951	DNA
26	contig00232:10901..11056 (-)	ACCGAGCTCGGCCACCATGGTGATGCTATGGATAGAATTTAA	CACACTGGACTAGTGGATCTACTTTTCCATCTTCTTTGCGA	156	DNA
27	contig00433:34958-36380(+)	ACCGAGCTCGGCCACCATGGTAGAGAATAAGAAGGT	CACACTGGACTAGTGGATCTATTCATCGAAATCTTTGTTTA	1422	DNA
28	contig00147:21973-23388(+)	ACCGAGCTCGGCCACCATGGTGATGACGGAAGCATTAAATAA	CACACTGGACTAGTGGATCTATGCTGCTAATGTTAAAAATG	1416	DNA
29	contig00145:9-1059(-)	ACCGAGCTCGGCCACCATGGTGATGAAAAATATAGTTGTTCT	CACACTGGACTAGTGGATCTAATCACCAACTAGATGATGAG	1050	DNA
30	contig00167:23417-24638 (-)	ACCGAGCTCGGCCACCATGGGTGAACAGCATGTAGAATCTAAAG	CACACTGGACTAGTGGATCTAACAACATTCCATATCTACAGGATTTG	690	cDNA
31	contig00063:31158-31905 (-)	ACCGAGCTCGGCCACCATGGTGAAATTCAATCCCAATGCAA	CACACTGGACTAGTGGATCTATAATGTTTTAGCTAGTAATGCATGCTC	759	cDNA
32	contig00062:98497-98689(+)	ACCGAGCTCGGCCACCATGGAATGCTTAGAATCCATAGG	CACACTGGACTAGTGGATCTAATTCAATATGGATATGTTCA	192	DNA
33	contig00079:101222-102015(+)	ACCGAGCTCGGCCACCATGGTGAAATATAGGGAGCAACGTAA	CACACTGGACTAGTGGATCTAGAAATAATTGAAATGGGGCT	795	DNA

Red=pVAX1 cassette sequence, blue=Kozak sequence, green=nucleotides for engineered stop codon (entire stop codon underlined)
*Candidate 32=Full length cf76, Candidate 33=C-terminal region of cf76. In addition to being in CF-Library, these two candidate comprise CF-1
^Not included in CF-Library (unable to amplify)
