# Supplementary material for: Identification of Cytauxzoon felis antigens via protein microarray and assessment of expression library immunization against cytauxzoonosis
Source: Clin Proteomics. 2018 Dec 29;15:44. doi: 10.1186/s12014-018-9218-9 (PMC6310948; doi:10.1186/s12014-018-9218-9)
Supplement: Supplementary file 3 — Additional file 3: Supplementary Fig. 1. Cytauxzoon felis DNA vaccination pilot study design. A total of 18 cats were divided into four groups assessed in this study: three cats vaccinated with CF-1 prior to infection (red box), four cats vaccinated with CF-Library prior to infection (blue box), eight cats that were not vaccinated prior to infection (green box), and three cats that were neither vaccinated nor infected (gray box). The eight unvaccinated, infected cats were involved in a separate study testing the efficacy of novel chemotherapeutics against cytauxzoonosis. If it became evident that vaccines or chemotherapeutics were not halting the progression of disease, cats were additionally given atovaquone and azithromycin to attempt to prevent death; these cats are noted accordingly. A&A = atovaquone and azithromycin, 4X ABX = pradofloxacin, doxycycline, clindamycin, and metronidazole. [file 12014_2018_9218_MOESM3_ESM.pdf]

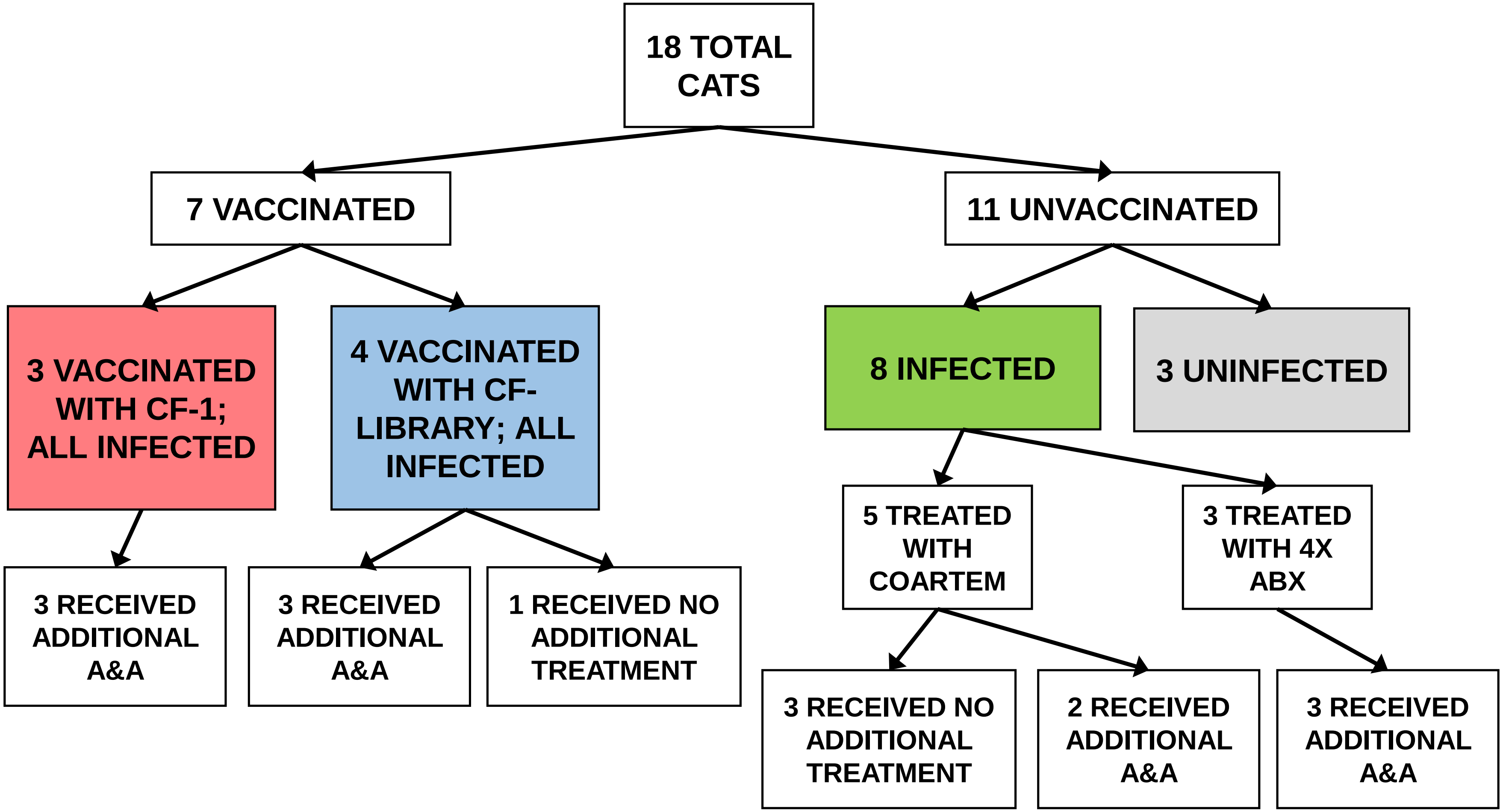

Supplementary Figure 1. *Cytauxzoon felis* DNA vaccination pilot study design. A total of 18 cats were divided into four groups assessed in this study: three cats vaccinated with CF-1 prior to infection (red box), four cats vaccinated with CF-Library prior to infection (blue box), eight cats that were not vaccinated prior to infection (green box), and three cats that were neither vaccinated nor infected (gray box). The eight unvaccinated, infected cats were involved in a separate study testing the efficacy of novel chemotherapeutics against cytauxzoonosis. If it became evident that vaccines or chemotherapeutics were not halting the progression of disease, cats were additionally given atovaquone and azithromycin to attempt to prevent death; these cats are noted accordingly. A&A=atovaquone and azithromycin, 4X ABX=pradofloxacin, doxycycline, clindamycin, and metronidazole.
