# Supplementary material for: Identification of Cytauxzoon felis antigens via protein microarray and assessment of expression library immunization against cytauxzoonosis
Source: Clin Proteomics. 2018 Dec 29;15:44. doi: 10.1186/s12014-018-9218-9 (PMC6310948; doi:10.1186/s12014-018-9218-9)
Supplement: Supplementary file 4 — Additional file 4: Supplementary Fig. 2. Timeline of vaccination, infection, and sample collection for vaccinated cats. [file 12014_2018_9218_MOESM4_ESM.pdf]

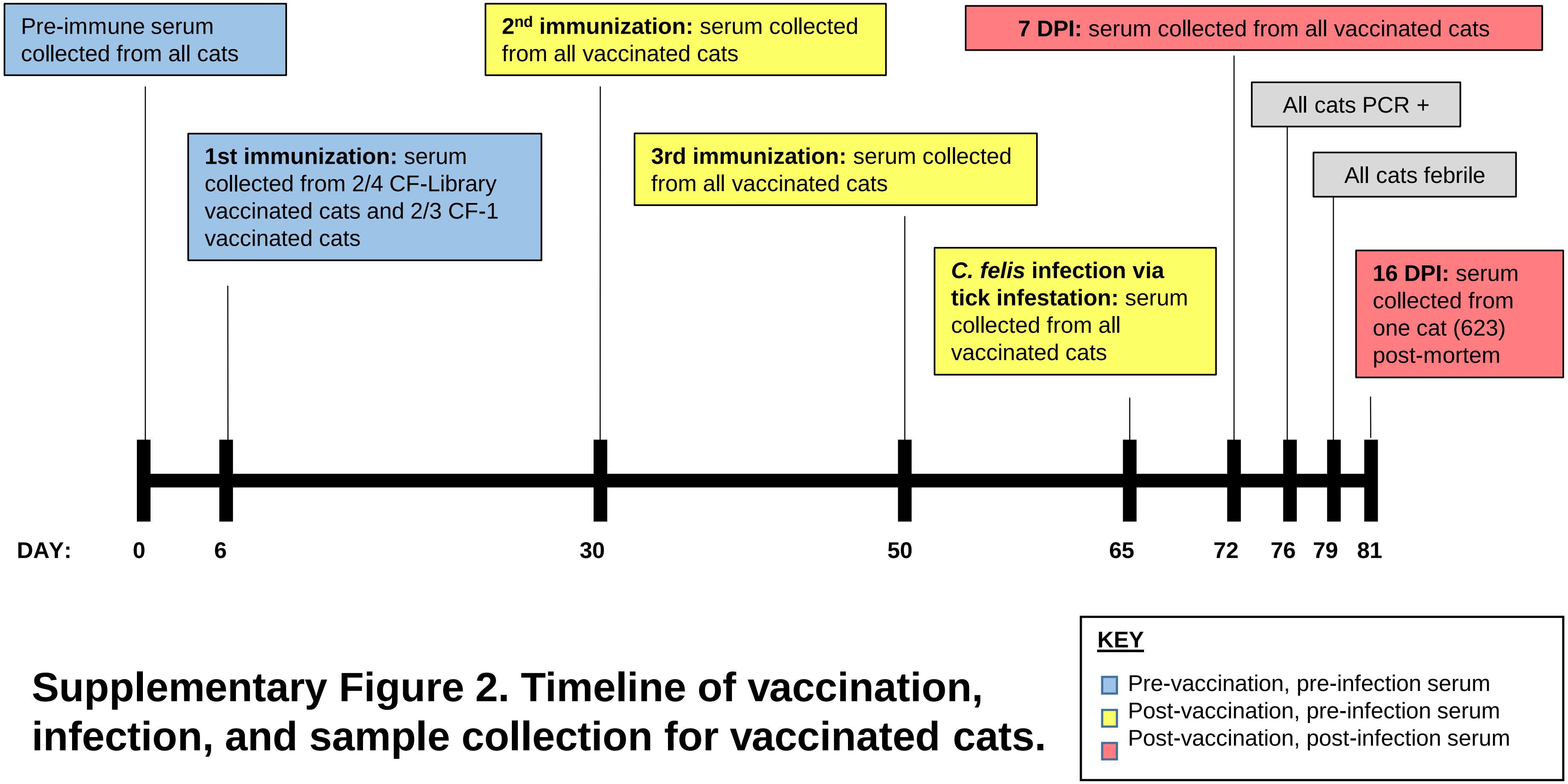

Supplementary Figure 2. Timeline of vaccination, infection, and sample collection for vaccinated cats.
