# Supplementary material for: Identification of Cytauxzoon felis antigens via protein microarray and assessment of expression library immunization against cytauxzoonosis
Source: Clin Proteomics. 2018 Dec 29;15:44. doi: 10.1186/s12014-018-9218-9 (PMC6310948; doi:10.1186/s12014-018-9218-9)
Supplement: Supplementary file 5 — Additional file 5: Supplementary Table 2. Additional clinical observations and information for individual cats. [file 12014_2018_9218_MOESM5_ESM.pdf]

Supplementary Table 2. Additional clinical observations and information for individual cats.

Test Group	Cat ID	Attached Ticks	Unattached Ticks	Total Ticks	Tick Comments	Initial Therapy	A&A Therapy Added	DPI febrile	DPI afebrile	DPI most severe illness	DPI improved
CF-Library	**77**	49	4	53	35 engorged; 18 unengorged	N/A	No	10	22	18	20
	**308**	43	3	46	1/2 engorged; 1/2 unengorged	N/A	Yes	12	19	17	19
	**339**	29	3	32	1/3 engorged; 2/3 unengorged	N/A	Yes	11	18	13	20
	**835**	45	1	46	1/4 engorged; 2/4 unengorged	N/A	Yes	10	16	15	20
CF-1	**331**	33	10	43	1/3 engorged; 2/3 unengorged	N/A	Yes	11	EUTHANIZED	16	EUTHANIZED
	**623**	50	4	54	1/4 engorged; 2/4 unengorged	N/A	Yes	13	EUTHANIZED	16	EUTHANIZED
	**638**	29	4	33	15 engorged; 14 unengorged	N/A	Yes	11	17	12	19
Unvaccinated,	**13**	44	3	47	Many big ticks, few engorged	4X Abx	Yes	11	17	13	18
Infected	**47**	56	0	56	Many engorged ticks	4X Abx	Yes	11	21	Unclear	22
	**59**	50	0	50	Lots of small ticks, few engorged	Coartem	No	12	19	18	21
	**84**	27	0	27	All small	Coartem	No	11	16	12	16
	**577**	52	1	53	N/A	4X Abx	Yes	12	16	18	EUTHANIZED
	**775**	47	3	50	Large ticks, unengorged	Coartem	No	11	17	12	17
	**797**	47	1	48	Lots of big ticks, some engorged	Coartem	Yes	12	18	18	FOUND DEAD
	**816**	45	6	51	Mostly small ticks, couple engorged	Coartem	Yes	13	17	18	20
Unvaccinated,	**264**	47	0	47	N/A	N/A	N/A	N/A	N/A	N/A	N/A
Uninfected	**276**	40	0	40	N/A	N/A	N/A	N/A	N/A	N/A	N/A
	**880**	47	0	47	N/A	N/A	N/A	N/A	N/A	N/A	N/A

A&A=atovaquone and azithromycin, 4X ABX=pradofloxacin, doxycycline, clindamycin, and metronidazole.
