# Supplementary material for: Identification of Cytauxzoon felis antigens via protein microarray and assessment of expression library immunization against cytauxzoonosis
Source: Clin Proteomics. 2018 Dec 29;15:44. doi: 10.1186/s12014-018-9218-9 (PMC6310948; doi:10.1186/s12014-018-9218-9)
Supplement: Supplementary file 6 — Additional file 6: Supplementary Table 3. Summary of supportive care administered to individual cats. Cats vaccinated with CF-Library received less overall supportive care. [file 12014_2018_9218_MOESM6_ESM.pdf]

Supplementary Table 3. Summary of supportive care administered to individual cats. Cats vaccinated with CF-Library received less overall supportive care.

		Categories								
**Test Group**	**Cat**	**Fluids**	**Anticoagulant**	**Analgesic**	**Antiemetic**	**Nutritional Support**	**Appetite stimulant**	**Transfusion**	**Other**	**Survival**
CF-Library	77	No	No	No	No	No	No	No	No	Alive
	308	Yes (SQ)	No	No	Yes	No	Yes	Yes (hyperimmune plasma)	Anticonvulsant (phenobarbital)	Alive
	339	Yes (SQ)	No	No	No	No	Yes	No	No	Alive
	835	Yes (IV + Bolus)	Yes	Yes	Yes	Yes	Yes	Yes (whole blood)	Antacid (famotidine)	Alive
CF-1	331	Yes (IV)	Yes	Yes	Yes	Yes	No	No	Ocular TAO + tropicamide*	Dead
	623	Yes (IV)	Yes	Yes	Yes	Yes	No	No	No	Dead
	638	Yes (SQ/IV)	Yes	Yes	Yes	Yes	No	No	No	Alive
Unvaccinated,	13	Yes (IV)	Yes	Yes	Yes	Yes	Yes	No	Zinc oxide on perineum^	Alive
Infected	47	Yes (IV)	Yes	Yes	Yes	Yes	Yes	Yes (whole blood)	Antibacterial (pradofloxacin)#, zinc oxide on perineum^	Alive
	59	Yes (IV)	Yes	Yes	Yes	Yes	Yes	Yes (hyperimmune plasma)	Antibacterial (pradofloxacin) #	Alive
	84	Yes (IV)	Yes	Yes	Yes	No	No	No	No	Alive
	577	Yes (IV)	Yes	Yes	Yes	Yes	Yes	No	Diuretic (furosemide)	Dead
	775	Yes (IV)	Yes	Yes	Yes	No	No	No	No	Alive
	797	Yes (IV + Bolus)	Yes	Yes	Yes	Yes	Yes	Yes (whole blood)	No	Dead
	816	Yes (IV)	Yes	Yes	Yes	Yes (+ Tumil K)	Yes	Yes (hyperimmune plasma)	Diuretic (furosemide)	Alive
Unvaccinated,	264	No	No	No	No	No	No	No	No	Alive
Uninfected	276	No	No	No	No	No	No	No	No	Alive
	880	No	No	No	No	No	No	No	No	Alive

Fluid therapy=crystalloid PlamaLyte with or without KCl, SQ=subcutaneous, IV=intravenous, Bolus=IV bolus of fluids administered in addition to baseline fluids, Anticoagulant=heparin (SQ or IV), analgesic=buprenorphine, antiemetic=maropitant, metoclopramide, and/or dolasetron, nutritional support=Clinicare, Tumil K=potassium supplement, appetite stimulant=mirtazapine, TAO=triple antibiotic ointment

*Used to treat blepharospasm in Cat 331

^Used to treat rectal bleeding and swelling in Cats 13 and 47

#Pradofloxacin administered independent of 4X Abx treatment regimen for Cat 47
