# Supplementary material for: Identification of Cytauxzoon felis antigens via protein microarray and assessment of expression library immunization against cytauxzoonosis
Source: Clin Proteomics. 2018 Dec 29;15:44. doi: 10.1186/s12014-018-9218-9 (PMC6310948; doi:10.1186/s12014-018-9218-9)
Supplement: Supplementary file 7 — Additional file 7: Supplementary Fig. 3. Serological responses to C. felis antigens are increased during chronic infection. Serologic reactivity of antigens are depicted as a heatmap in (A). Antigens are listed in rows while grouping of individuals are denoted in columns. Average signal intensity of individual antigens against serum from acute and chronic groups are depicted in (B). The majority of antigens that had a higher average reactivity against serum from chronically infected cats (n = 50), but two antigens were more reactive against serum from acutely infected cats (n = 2, denoted with yellow boxes). These two antigens were incorporated into the CF-Library vaccine as Candidates 32-33. [file 12014_2018_9218_MOESM7_ESM.pdf]

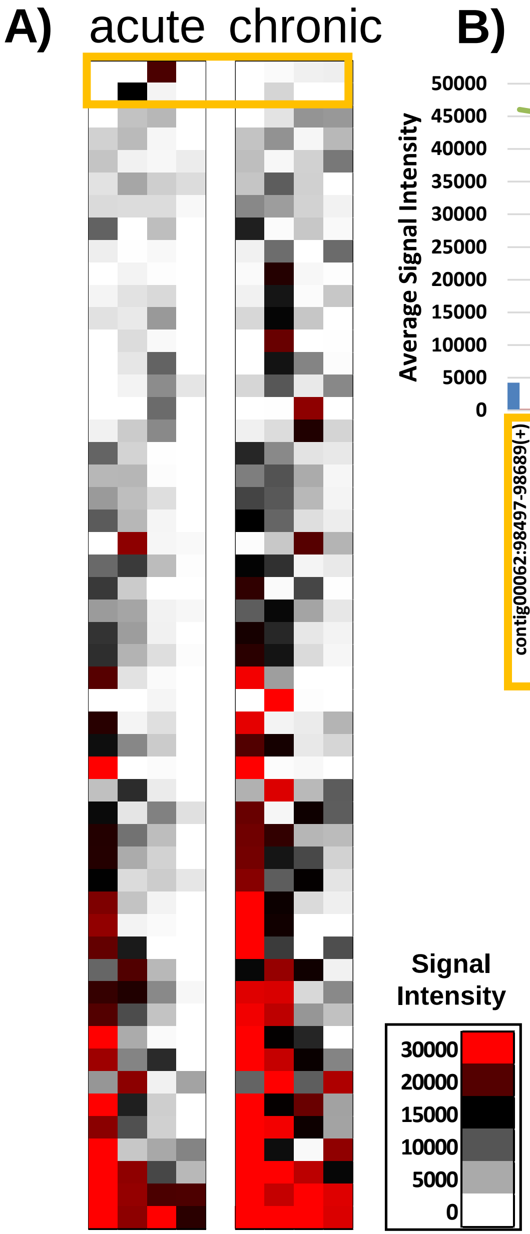

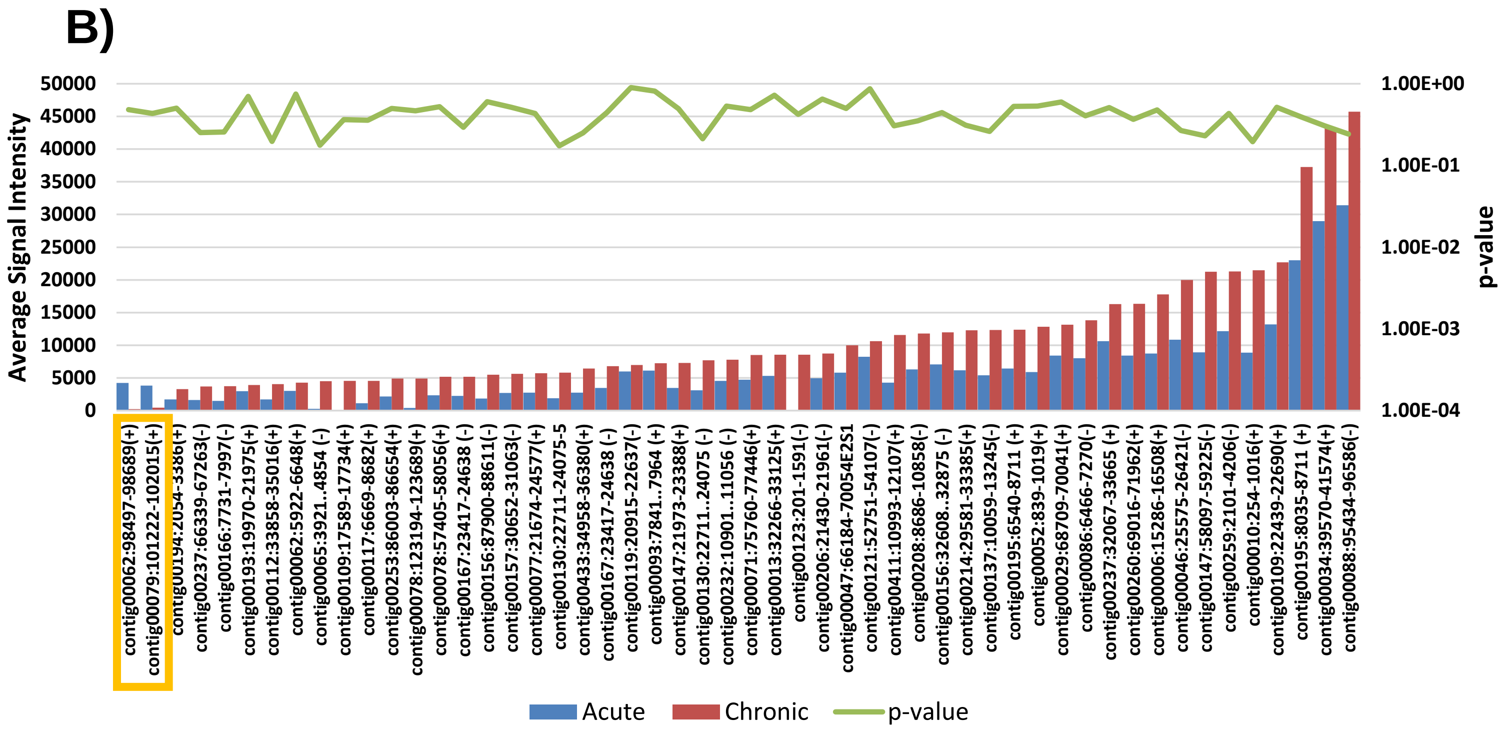

Supplementary Figure 3. Serological responses to *C. felis* antigens are increased during chronic infection. Serologic reactivity of antigens are depicted as a heatmap in (A). Antigens are listed in rows while grouping of individuals are denoted in columns. Average signal intensity of individual antigens against serum from acute and chronic groups are depicted in (B). The majority of antigens that had a higher average reactivity against serum from chronically infected cats (n=50), but two antigens were more reactive against serum from acutely infected cats (n=2, denoted with yellow boxes). These two antigens were incorporated into the CF-Library vaccine as Candidates 32-33.
