# Supplementary material for: Identification of Cytauxzoon felis antigens via protein microarray and assessment of expression library immunization against cytauxzoonosis
Source: Clin Proteomics. 2018 Dec 29;15:44. doi: 10.1186/s12014-018-9218-9 (PMC6310948; doi:10.1186/s12014-018-9218-9)
Supplement: Supplementary file 8 — Additional file 8: Supplementary Fig. 4. Serological profiles of individual vaccinated cats to candidates throughout study. Arrays containing the 32 vaccine candidates included in CF-Library and in CF-1 were probed with sera samples from individual cats. Heat map shows normalized signal intensity with red strongest, white weakest, and gray intermediate. Rows denote 32 different candidates included in vaccines listed in descending order of reactivity; candidates 19 and 3 were included in CF-1, while all listed candidates were included in CF-Library. Results are organized by individual cats (identified by number at top), and survival status of each cat is indicated as “A” (alive) or “D” (dead). Individual columns within each cat’s array represent serum samples collected at different time points through study (labeled by the day in the study the sample was collected; refer to Supplemental Fig. 2 for timeline). There was no correlation between immunization protocol, individual reactivity, and survival for most cats, with the exception of Cat 77, who had widespread yet weak reactivity against all candidates in the CF-Library vaccine prior to infection and subsequently had milder disease and did not require supportive care or antiprotozoal therapy. [file 12014_2018_9218_MOESM8_ESM.pdf]

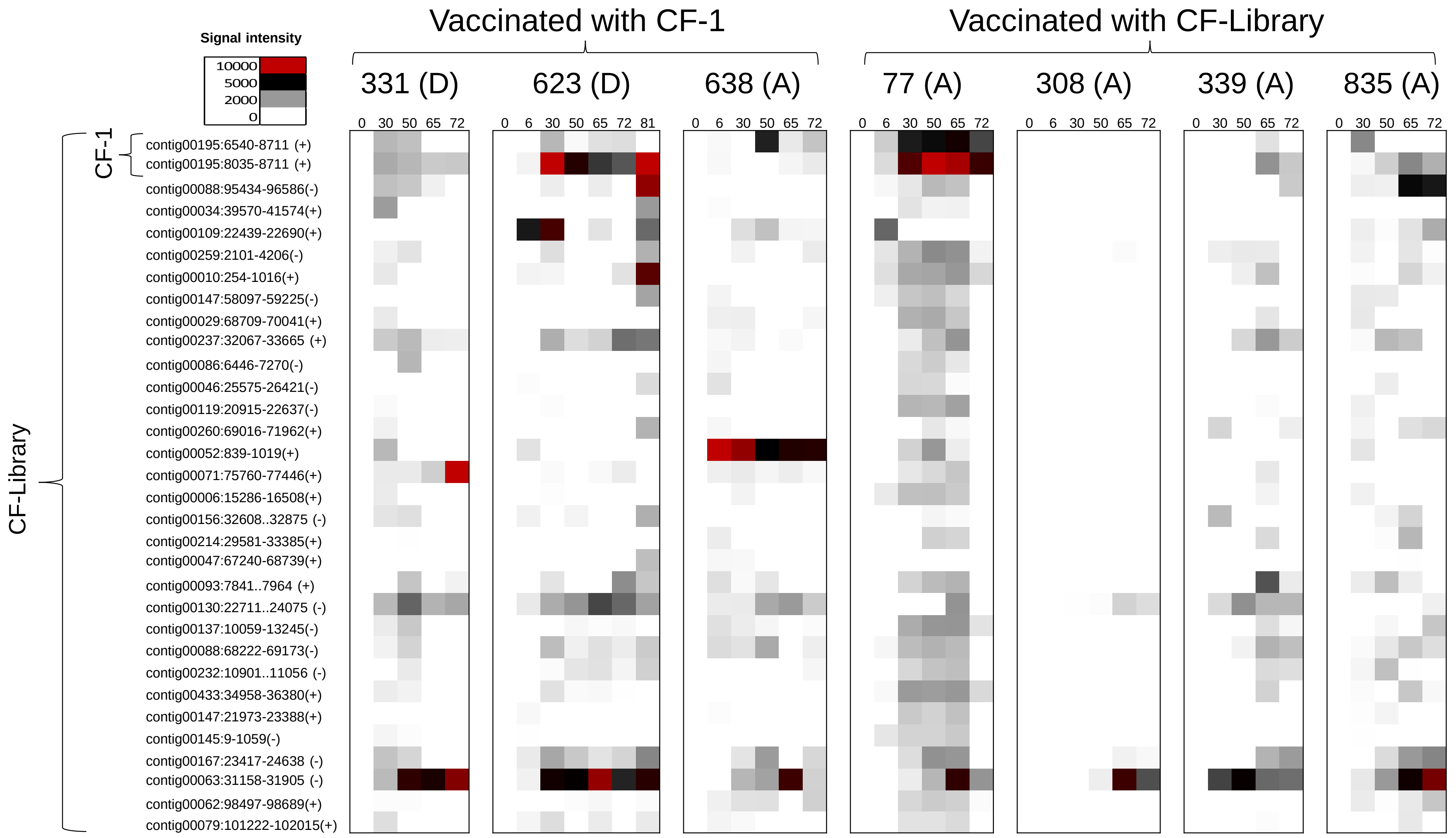

Supplementary Figure 4. Serological profiles of individual vaccinated cats to candidates throughout study. Arrays containing the 32 vaccine candidates included in CF-Library and in CF-1 were probed with sera samples from individual cats. Heat map shows normalized signal intensity with red strongest, white weakest, and gray intermediate. Rows denote 32 different candidates included in vaccines listed in descending order of reactivity; candidates 19 and 3 were included in CF-1, while all listed candidates were included in CF-Library. Results are organized by individual cats (identified by number at top), and survival status of each cat is indicated as “A” (alive) or “D” (dead). Individual columns within each cat’s array represent serum samples collected at different time points through study (labeled by the day in the study the sample was collected; refer to **Supplementary Figure 2** for timeline). There was no correlation between immunization protocol, individual reactivity, and survival for most cats, with the exception of Cat 77, who had widespread yet weak reactivity against all candidates in the CF-Library vaccine prior to infection and subsequently had milder disease and did not require supportive care or antiprotozoal therapy.
